# A codevelopment process to advance methods for the use of patient‐reported outcome measures and patient‐reported experience measures with people who are homeless and experience chronic illness

**DOI:** 10.1111/hex.13489

**Published:** 2022-04-11

**Authors:** Erin E. Donald, Kara Whitlock, Tracy Dansereau, Daniel J. Sands, David Small, Kelli I. Stajduhar

**Affiliations:** ^1^ Institute on Aging and Lifelong Health University of Victoria Victoria British Columbia Canada; ^2^ Faculty of Human and Social Development, School of Nursing University of Victoria Victoria British Columbia Canada

**Keywords:** health equity, homeless, homelessness, patient‐oriented research, patient‐reported outcomes, substance use

## Abstract

**Introduction:**

People who experience social disadvantage including homelessness suffer from numerous ill health effects when compared to the general public. Use of patient‐reported outcome measures (PROMs) and patient‐reported experience measures (PREMs) enables collection of information from the point of view of the person receiving care. Involvement in research and health care decision‐making, a process that can be facilitated by the use of PROMs and PREMs, is one way to promote equity in care.

**Methods:**

This article reports on a codevelopment and consultation study investigating the use of PROMs and PREMs with people who experience homelessness and chronic illness. Data were analysed according to interpretative phenomenological analysis.

**Results:**

Committee members with lived experience identified three themes for the role of PROMs and PREMs in health care measurement: trust and relationship‐building; health and quality of life; and equity, alongside specific recommendations for the design and administration of PROMs and PREMs. The codevelopment process is reported to demonstrate the meaningful investment in time, infrastructure and relationship‐building required for successful partnership between researchers and people with lived experience of homelessness.

**Conclusion:**

PROMs and PREMs can be meaningful measurement tools for people who experience social disadvantage, but can be alienating or reproduce inequity if they fail to capture complexity or rely on hidden assumptions of key concepts.

**Patient or Public Contribution:**

This study was conducted in active partnership between researchers and people with experience of homelessness and chronic illness, including priority setting for study design, data construction, analysis and coauthorship on this article.

## 1 INTRODUCTION

Homelessness is defined as ‘living in a shelter, public space, abandoned vehicle, or someone else's home’.^1^ ^p.578^ There is considerable overlap and movement between homelessness and unstable housing, a situation in which a person or family is housed, but has experienced multiple moves over the past year.^1^ Internationally, 1.6 million people experience inadequate housing, with an estimated 400,000 of those located in Canada.^1^, ^2^ People experiencing homelessness suffer from death rates 2–5 times higher than the general public for the same causes, have worse physical and mental health, have more chronic conditions, experience higher rates of traumatic brain injury and substance use and experience earlier declines in health typically associated with ageing.^2^, ^3^

Worse physical and mental health is coupled with an unmet need for care.^1^, ^3^ One study of unmet health need found no significant differences between people who were homeless and vulnerably housed, suggesting that these groups share similar health states and challenges accessing care.^1^ There are many barriers to care including lack of transportation, lack of child care, long waitlists, competing priorities for survival, inaccessible care settings (such as those with abstinence‐only drug policies) and discrimination in the health care system.^1^, ^4^, ^5^ Even in countries with policy mandates for universal access to care, divisions of responsibility between care sectors, and between health and social services, can lead to people ‘falling between the cracks’.^4^, ^5^

People experiencing homelessness and vulnerable housing are impacted by both individual and structural circumstances, resulting in social disadvantage.^6^ Socially disadvantaged groups experience poverty, discrimination, stigmatization and marginalization that impact access to health care and inclusion in health care research.^7^ These factors limit choices and opportunities, increasing the risk of harm from other individuals and social structures.^4^ Social disadvantage shifts over time in response to changes in external social forces and their impact on intersecting identities such as disability status, indigeneity and sexual or gender orientation.^4^, ^8^, ^9^, ^10^ In Canada, where Indigenous people are up to eight times more likely to be homeless or precariously housed compared to nonindigenous Canadians, historical and ongoing colonization has been particularly destructive to the health of Indigenous people. This has resulted in ongoing inequalities in health and social positioning to this day.^11^, ^12^, ^13^

Despite these impacts, it is important to recognize the vibrant strength and resilience inherent in people who are experiencing housing issues and in the communities and relationships formed in these contexts.^4^, ^14^ Though often overlooked, this community strength and resilience is a powerful source of expertize and insight for researchers and organizations working in these contexts.

Inclusion in health research and decision‐making is vital to reducing inequities in the health care system.^6^ When researchers, policymakers and health care providers (providers) base their decision‐making on research that has excluded people who are socially disadvantaged, the results are likely to reproduce inequity.^4^, ^6^ One way of including what matters to people who are socially disadvantaged in both research and health care is the use of patient‐reported outcome measures (PROMs) and patient‐reported experience measures (PREMs). PROMs and PREMs are measurements of health status, quality of life, experience and satisfaction from the point of view of the person receiving care and their family.^15^, ^16^ This paper reports on the codevelopment and initial consultation study investigating the use of PROMs and PREMs with people who experience social disadvantage, as reported jointly by members of a committee of people with lived experience of homelessness and researchers. To inform this study and in the context of lived experience of homelessness, we joined together to ask, ‘What is most important to measure about our health care, and how can it best be measured using PROMs and PREMs?’.

## 2 METHODS

This study used interpretative phenomenological analysis (IPA) with a codevelopment framework.^17^, ^18^ Codevelopment is both a method and a philosophy that involves research activities done *with* or *by* members of a group instead of *about* or *for* them.^19^, ^20^ The aim is to authentically involve stakeholders in all phases of research, including the ongoing commitment to work together for change.^20^, ^21^ Joint efforts between stakeholders can strengthen the influence of research through collective impact.^21^ When members of the public are involved in research testing and designing the use of tools like PROMs and PREMs, these tools can become clearer, more acceptable and better meet the needs of the people who will use them.^7^, ^22^

The codevelopment process is part of a larger study funded by the British Columbia SUPPORT Unit Patient‐Centred Measurement Methods Cluster, part of Canada's Strategy for Patient‐Oriented Research, with the overall aim of advancing methods in the use of PROMs and PREMs with people who are socially disadvantaged and who experience chronic illness. The study is part of a larger multiyear programme of research that relies on and invests in relationships with a local inner‐city community and service organizations serving people who are socially disadvantaged.^4^, ^23^, ^24^, ^25^ Ethical approval was obtained from the University of Victoria. Alongside other relevant stakeholders including clinicians and a community health centre, the authors make up the People With Lived Experience Advisory Committee (the committee) engaged as expert consultants and coknowledge producers throughout the study. K. W. and K. S. were co‐chairs of the committee. K. W. is a research assistant with lived experience. T. D., D. J. S. and D. S. are expert contributors, sharing their own lived experience and expertise. All committee members with lived experience have a history of homelessness and chronic illness, including but not limited to substance use. E. D. is a doctoral candidate and coordinator for this phase of the project, and K. S. is the principal investigator. The grant was crafted with input from an action team involved in K. S.'s programme of research and included roles for the committee at each stage.^6^, ^7^, ^21^, ^26^

### 2.1 Engagement process

The committee co‐chairs originally planned to reach out to patient advisory groups from inner‐city organizations and extend open invitations to join the committee,^27^ but just as they were starting, the COVID‐19 pandemic hit North America. For a community already facing rampant social inequity, COVID‐19 compounded existing crisis conditions. Community partners no longer had the capacity to support research, patient advisory groups shut down and new restrictions prohibited face‐to‐face meetings. K. W. reached out to existing organizational partners working in the inner city to ask how we might best shift our engagement process considering these emerging restrictions and additional burdens facing potential members. With the awareness that this would limit who we could successfully engage, and recognizing that community‐based research with people who face social disadvantage requires flexibility and reprioritization based on community need,^14^, ^20^, ^21^ the committee co‐chairs chose to pursue the suggestion of smaller virtual video meetings with an advisory committee consisting of people who had lived experience of homelessness but were now housed in stable living situations and had access to equipment and internet services that would allow for full video participation. Although the research team recognized that this meant that our research may not capture the perspectives that we had originally hoped, flexibility in community research requires the research agenda to adapt to community capacity.^26^ This flexibility is demonstrated by shifting our gaze from what we had hoped to do (i.e., engage in face‐to‐face focus groups in community alongside building an ongoing advisory committee of 8–10 people living with social disadvantage) to what was possible (i.e., a smaller virtual advisory committee with experience of homelessness, but who were now housed).

K. W. reached out through existing relationships and networks^7^ to invite a diverse group to participate. Initially, recruitment posters were distributed to, and posted on bulletin boards of, inner‐city organizations, describing the study and inviting potential participants to contact the research team. When services were disrupted due to COVID‐19, the team adapted their recruitment approach and relied on previous research relationships with inner‐city service providers to distribute the posters to clients they had relationships with. Potential participants were recruited for participation on the advisory committee if they self‐identified as a person with experiences of poverty and homelessness and/or health care discrimination, were >18 years of age and spoke English.

Over the 10‐week recruitment period, six potential participants with lived experience responded to the recruitment advertisement. K. W. called and screened each of the six potential participants for inclusion and participation in the advisory committee. While all six potential participants fulfilled the inclusion criteria and expressed interest in participating, three people left or did not attend the initial two meetings despite multiple attempts to follow up by phone and email. While we are unclear why this occurred, it is likely that the increased burden in the context of multiple health emergencies facing the inner‐city community may have limited some people's capacity to participate. By the third meeting, participation had stabilized to six members, three with lived experience of homelessness and chronic illness, one member with lived experience and a researcher role and two members of the research team without lived experience. The committee met eight times by video conference between May 2020 and August 2020. Committee members with lived experience were compensated $25 per hour for their time and expertise.^27^

Taking direction from Boilevin and colleagues^27^ on conducting ethical research with vulnerable populations, the research team intentionally built in time to develop the trust necessary for this experiential exploration. One way in which we built this relationship was to encourage authentic sharing by all group members, especially from the research team, to disrupt traditional power dynamics embedded in research relationships.^27^, ^28^ This practice aligns with recommendations that researchers approach community members with the willingness to share about themselves and their own positionality, with the same openness being asked of community partners.^20^, ^27^

Another important process was beginning with reflexivity, defining our own identities and experiences.^20^ For example, D. J. S. introduced themselves by presenting their own positioning, explaining where they came from, how they saw themselves and who they were in the context of the world around them. Although D. J. S. is providing their own perspective of the needs and experiences of people with lived experience of homelessness and chronic illness, this location is not static.^20^

The first five meetings included collaborating on the committee objectives, drafting our guidelines for group process (see Appendix SA) and defining terms relevant to PROMs and PREMs (e.g., measurement, health care) in the context of homelessness and chronic illness. The committee spent these meetings exploring our experiences in relation to health and health care services, as well as describing how these were impacted (or not) by shifting circumstances. For each meeting, K. W. and E. D. prepared discussion prompts guided by the research question and designed to elicit an insider's perspective on meaning making in relation to PROMs and PREMs^17^, ^28^ (see Appendix SB). These prompts were seldom required after the meeting began, as participants with lived experience played an active role in guiding the conversation.

For the final three meetings, the committee focused on identifying and refining these concepts of PROMs and PREMs through discussions of how they might be demonstrated in practice for people experiencing homelessness and chronic health issues. For example, the concept of trust with a health care provider was described as the provider actions that have inspired greater or lesser trust for committee members. Moving vague experiential concepts into a tangible realm was often the impetus for deeper conversation as people articulated the meaning(s) and interpretations of lived experiences.

Finally, as concepts were explored and refined, the group transitioned to discussing examples of commonly used PROMs and PREMs, including the Veterans Rand 36‐Item Health Survey (VR‐36), the Veterans Rand 12‐Item Health Survey (VR‐12) and the World Health Organization Quality of Life—Abbreviated (WHOQoL‐BREF).^30^, ^31^ Committee members provided feedback on the suitability of these tools and used them as a launching point for discussion about facilitators and barriers to tool administration. Collectively, these meetings generated a series of themes, as well as specific recommendations for the design and delivery of PROMs and PREMs.

**Table 1:** Recommendations for the design and implementation of PROMs and PREMs

Recommendation	Rationale	Examples
Questionnaires should be designed with accessibility in mind	This includes traditional measures of accessibility for people who experience a range of disabilities and chronic illnesses, but also for those who are using substances, live with memory loss, experience symptoms of mental or physical distress or who experience challenges reading	Accessibility may include large font, translations, verbal administration, additional time for completion and many other strategies. For implementation by researchers, policymakers or decision‐makers at local clinical sites who are designing PROMs and PREMs for use in practice.
Demographic data should be as inclusive as possible, with the ‘tick all boxes that apply’ format and a wide range of options	People who are homeless or vulnerably housed with chronic illnesses often experience intersecting marginalized identities (racial identity, sexuality, etc.). Tool designers and administrators can be more inclusive by being as flexible as possible when asking people to self‐identify	Include ‘tick all boxes that apply’ options rather than forced choice. Include a wide range of demographic options. Where possible, allow respondents to provide their own language for categories such as gender, sexuality, religion/spirituality, etc. For implementation by researchers, policymakers or decision‐makers at local clinical sites who are designing PROMs and PREMs for use in practice.
Use multiple options for scales, or simple scales where possible	Committee members were divided on preference for visual, numeric or written response scales. This demonstrated a variety of preferences and that different scales are more accessible to different respondents	For example, when rating satisfaction, use of a visual analogue scale alongside a written or numeric scale. For implementation by researchers, policymakers or decision‐makers at local clinical sites who are designing PROMs and PREMs for use in practice.
Have a ‘not applicable’ option for all questions	Being forced to provide an answer to a question that does not apply can be frustrating and alienating. Like providing as many demographic options as possible, ‘not applicable’ options for answers allow respondents to ensure that their participation is more reflective of their experience	Include a not applicable option for all answers. For implementation by researchers, policymakers or decision‐makers at local clinical sites who are designing PROMs and PREMs for use in practice.
Clarify a limited time range	To qualify for social services, treatment coverage and access to specialized clinics (such as those that accept people who are homeless or vulnerably housed), people are frequently expected to recount their life stories including reliving multiple instances of trauma	Ask about a limited time range to avoid misunderstanding. For implementation by anyone designing or administering a PROM or PREM.
Ask about time ranges in ways that are relevant to a person's experience	Memory issues, either permanent or temporary, are a common access barrier. For example, a person may not be able to remember 6 months ago, or may not be able to align their experience with months of the year	Use prompts such as ‘for this visit’, ‘since you started seeing me’ or ‘compared to your best/worst day?’ to be more accessible for those with memory issues. For implementation by anyone designing or administering a PROM or PREM.
Ask about perceived ability and barriers to accessing care	Many PROMs and PREMs are designed for populations who do not experience significant barriers to accessing care. For people who are homeless or vulnerably housed with chronic illness, it is an indispensable component of both health care experience and outcome. These responses will also be important for any provider, organization or researcher who is interested in making care more accessible and collecting information about who may not be able to access care	Include questions about perceived ability to access care and perceived barriers to care on PROMs and PREMs. For implementation by researchers, policymakers or decision‐makers at local clinical sites who are designing PROMs and PREMs for use in practice.
Tools should be generic enough to use across sites and between professional roles	People who are homeless or vulnerably housed with chronic illness form care relationships in a variety of settings with people in different roles. A person's primary provider may not be immediately visible or expected	Avoid provider‐ or role‐specific language in the design of PROMs and PREMs. For implementation by researchers, policymakers or decision‐makers at local clinical sites who are designing PROMs and PREMs for use in practice.
Experience measures are equal to or more important than outcome measures. An ideal approach would measure both	PROMs and PREMs depend on a trusting relationship between the person and the provider/organization for engagement and authenticity. People who are homeless or unstably housed, and those with stigmatized chronic illnesses, are more likely to have negative experiences in the health care system. Experience measures can build trust by demonstrating the importance of respecting clients' dignity.	Include a combination of evaluation and outcome measures when designing and selecting evaluation tools for this population. For implementation by researchers, policymakers or decision‐makers at local clinical sites who are designing or selecting PROMs and PREMs for use in practice.
Emphasize that there are no wrong answers and no penalty for answering honestly	With few options to switch providers and little power in the health care system, people may be wary of the risk of losing access to care or receiving substandard care if they provide negative feedback	Include standardized language in the design of PROMs and PREMs. However, standardized language is not a substitute for a trusting relationship. Some techniques may include administration of PROMs and PREMs by third parties (not providers), periodic re‐evaluation using PROMs and PREMs as relationships develop and establishing a relationship before introducing a PROM or PREM. To be implemented by researchers, decision‐makers and practitioners who are using PROMs and PREMs in research or practice.

Abbreviations: PREM, patient‐reported experience measure; PROM, patient‐reported outcome measure.

### 2.2 Analysis

Analysis was guided by IPA, an approach that combines multiple philosophies to produce a nuanced exploration of lived experience and the meaning that people make of their lives.^18^, ^29^, ^32^ Originally proposed by Smith^17^ for the psychology of health care, IPA has since been adopted by researchers across disciplines, including nurse researchers in partnership with service users.^33^ By promoting ‘…an interpretive process between the researcher and the researched…’,^29^ IPA is particularly well suited to the investigation of under‐researched phenomena and to research in partnership.^33^

Meetings were recorded and transcribed. E. D. and K. W. jointly conducted the initial data analysis by reading and rereading transcripts, recording their reflections in the margins and meeting frequently to discuss their interpretations in an active hermeneutic process.^29^, ^31^ By including one team member with lived experience of homelessness and chronic illness (K. W.) and one researcher (E. D.) in this initial stage of analysis, K. W. and E. D. benefitted from each other's insight while reinforcing reflexivity that investigated and challenged both researcher privilege and the potential projection of personal experience on findings.^27^, ^32^ This inclusion of a perspective of lived experience in the initial stages of our analysis allowed for more meaningful comparisons and a more complete understanding of findings. As we explored the differences in our interpretations of the advisory committee process, we came to recognize how our personal frames of reference exposed the tensions that emerged from the measurement of multiple perspectives and priorities across different levels of health care. For example, E. D.'s broad population measurement lens contrasted with and complemented K. W.'s focus on the individual point of measurement. Bridging microlevel perspectives of care to broader macrolevel performance measures of health systems became critical to the creation of our shared understanding. As Kwon et al.^34^ state, we can benefit from acknowledging the role of these tensions in our analytic process. When E. D. and K. W. produced a set of proposed themes, they returned these to the committee as a whole for discussion and feedback.^29^ This acted as a form of member checking and prompted the creation of a plain language summary and report back to the full research team jointly produced by all committee members.

## 3 RESULTS

Three themes emerged from conversations about PROMs and PREMs. These are trust and relationship‐building; health and quality of life; and equity. The committee proposed specific recommendations for the design and administration of PROMs and PREMs with people who experience social disadvantage, comprising a fourth category of findings (see Table 1 for these recommendations).

### 3.1 Trust and relationship‐building

Trust and relationship‐building was the first and most important concept identified for inclusion in health care measurement. A trusting relationship with both individuals and organizations was deemed crucial to all good health care and to the implementation of PROMs and PREMs. Trust was understood to be multidimensional, with organizations, providers and persons receiving care all having a role in relationship‐building. However, these relationships are built in a system with severe power imbalances, where persons receiving care have little recourse against mistreatment or stigma. When organizations and providers incorporate feedback to change or improve systems of care, trusting relationships are possible. PROMs and PREMs can facilitate this process.

Factors that contribute to developing a trusting relationship include taking the time to listen, treating people with respect and acknowledging shared humanity. Additional important factors include access to shared decision‐making, resolving conflict and understanding the vulnerability inherent in seeking care. When committee members felt cared for, it opened the door to feeling heard and believed. This was described as a necessary ingredient for good care over all eight meetings and was a rare experience across health settings. When persons receiving care did not feel heard and believed, or when they felt shamed, dismissed or stigmatized, this resulted in unaddressed care needs and eventual care avoidance:

*You know, you're not getting tested for this as often as you should because you're afraid to go to the doctor because the doctor will look down his nose at you and make you feel bad about yourself, make you feel uncom‐ you know, you're going to feel all these things from the doctor, so you won't go. So you don't get your blood works or your whatever or your medications renewed as often as you should. And that affects your overall health*.

Being believed, heard and accepted was not just imperative for relationship‐building; committee members suggested that it was a crucial component to consider in the design and implementation of PROMs and PREMs. Members shared that when providers did not believe them or did not have an understanding of their circumstances, they were more likely to receive poor care and experience a cascade of harm. These experiences of poor care were explicitly linked to their consideration of outcome measures. For example, throughout our codevelopment process, committee members repeatedly expressed a desire for PROMs and PREMs that included separate dimensions for measuring the establishment of trust and relationship‐building in their interactions with providers. Committee members suggested that these core competencies would be a precursor to them even contemplating whether they would engage in the completion of PROMs and PREMs in clinical settings.

Committee members stressed the importance of ensuring that care would not be negatively impacted by a person's response to a PROM or PREM, and of explicitly communicating this to persons receiving care (see Table 1). Prompt and respectful responses to feedback provided via PROMs and PREMs can reinforce relationships and build trust.

### 3.2 Health and quality of life

Health was described by advisory committee members as a multidimensional concept including physical, mental, spiritual, cultural, social and ecological components. While physical and mental health were understood to be addressed primarily addressed by the health care system, committee members were explicit about the importance of accessing social connections, community involvement and safe and pleasant housing as factors that significantly influenced their health.

Health was understood to be a fluctuating state that could easily be impacted by the ability (or inability) to respond with agency to life events. Intersecting marginalization, minority identities and history of trauma(s) had a compounding effect on health, but committee members emphasized that paternalistic assumptions about identity and trauma could erase their individual circumstances and autonomy when wielded in care settings.

Quality of life encompassed all of these dimensions of health and the ability to live well according to one's own values. Quality of life included having one's needs met; having choices; and having a purpose, a hope or something to live for:

*So for me personally, it's being able to look after myself, as much freedom to do as much as I can with my person, whether it's physical limitations, mental limitations, addiction limitations*.

Advisory committee members agreed that PROMs and PREMs measuring multiple dimensions of health, quality of life and experience in the care setting could be effective ways to capture this complexity.

### 3.3 Equity

PROMs and PREMs were seen by committee members as a powerful tool for data collection at both the individual and system level and as a way to capture individualized data in a standardized framework that could help ensure equitable care. As one member stated:

*You go to a doctor with pain, you get nothing because you were a drug addict. Not ARE a drug addict, WERE a drug addict. They might relapse, they might start using [again] … It's ludicrous. The whole thing is insane. If a questionnaire, generic questionnaire to patients to get some sort of grip on what's happening … that would be friggin' amazing*.

Committee members described having unique care needs due to their experiences of homelessness, substance use or chronic illness, but reported having little choice or control over their care. Committee members were informed about their own health and had clearly identified preferences and values for their care, which they reported were not always valued by providers. If a committee member wanted to change a course of treatment, refuse treatment or switch providers, they were often restricted in doing so based on the limited services available, restrictive policies, limited financial resources or due to stigma and lack of providers with relevant expertise.

Committee members reported that providing feedback on a questionnaire such as a PROM or PREM could mean risking their care. Members described many instances of having to choose between accepting poor treatment or forgoing care entirely. While some of this is rooted in a relationship with an individual provider or site, systemic factors play a powerful role. Committee members described how forms that demonstrate inclusivity had influenced their impression of an organization or set the tone for a health care encounter:

*I think, you know, if I can see myself in, that I'm reflected or represented in this form, I'm going to be much more involved and engaged with it and more honest, right?*

According to committee members, PROMs and PREMs have the ability to convey risk or safety in their design. Clarity and accessibility were key to committee members' recommendations (see Table 1).

## 4 DISCUSSION

These findings offer insight into the complex health experiences of committee members in the context of PROMs and PREMs. In discussions of trust and relationship‐building, committee members were clear that while individual relationships with health care providers were important, a broader trust in organizations and systems impacted the delivery of care. These findings echo those presented by Treloar et al.,^35^ whose interviews with staff and clients at syringe programmes describe a multidimensional trust‐building process that relies not only on personal relationships but on organizational actions and reputation built over an extended period of time. Committee members had countless experiences of poor care before, during and after their time on the street, and had little trust in the health care system. For committee members, accessing the health care system was a completely different experience than that of the general public who were involved in studies designing commonly used PROMs and PREMs.^36^ Any assumptions of trust and relationship with the health care system that are inherent in the design of existing PROMs and PREMs should be in the minds of researchers and organizations who are interested in using these tools with people who are socially disadvantaged.

In relation to the design and implementation of PROMs and PREMs, it is not enough to elicit feedback. A trusting relationship requires feedback to be acted upon, an idea that continues to be met with some debate and resistance.^37^, ^38^ For committee members, both providers and organizations need to be listening to PROMs and PREMs for these tools to have meaning. Consideration should be given not only to how PROMs and PREMs are traditionally used as overall group measures but also how they might be used to reflect individual outcomes and experiences that could guide clinical encounters between providers and people who experience social disadvantage.

Committee members experience health and quality of life in holistic, multidimensional ways. Despite Canada's fragmented health and social services systems, committee members understood health to incorporate a range of social and systemic health determinants including housing, income, community connection and spirituality. In this way, committee members pushed back against Canada's colonial isolation of health services that divide the disease‐focused emphasis of the health care system from the social spaces and areas of policy impact where health is shaped.^39^, ^40^ Hubley et al., in their recent review of literature on subjective quality of life in homelessness, found few studies that had conducted analyses of individual domains or how changes in social circumstances or health status were related to change in subjective quality of life over time.^41^ While Hubley et al.^41^ recommend further research using standardized tools, tools developed for the general public may not identify differences relevant to the lives of people who are socially disadvantaged and we are encouraged by this same research team's development of population‐specific measures involving input from people with lived experience.^42^

Committee members believed that well‐designed PROMs and PREMs, when used for health service accountability, development and evaluation, have the ability to improve care. This might mean making the needs of people who are socially disadvantaged visible to providers and policymakers, or allowing for a person's health status to be compared objectively to other population members. However, PROMs and PREMs can be alienating and perpetuate inequity if they fail to capture complexity, assume a shared understanding of concepts^22^ or locate health inequities within the individual rather than within the individual's access to care and determinants of health.

Committee members believed that PROMs and PREMs must be codeveloped alongside people with lived experience and should move beyond being sensitive to being representative, a position supported by Neale and Strang.^22^ Meaningful codevelopment and participatory‐based research allows for increased access, richer data and deeper analysis while providing opportunities to strengthen communities and community partners.^21^, ^43^ As Wiering et al.^44^ point out, if we are to truly capture the patient's perspective, it is essential that they are involved in the development of measures that are most meaningful to them. Engaging people with social disadvantage in the development of PROMs and PREMs, and in the design of how they are implemented, is therefore necessary to ensure that outcomes and experience measures are representative of their perspective, and that measures are relevant, meaningful and valid. However, involving people experiencing social disadvantage in research does not guarantee meaningful participation. If research engagement is mere tokenism, it can lead to frustration, disillusionment with research and harm relationships with the community.^26^, ^43^ Research can be helpful, but it has also caused harm by perpetuating stigma, increasing inequality, exploiting pain and exhausting community resources.^20^, ^26^, ^27^ Key facilitators to respectful and reciprocal research include openness, authentic listening, investing time for trust and capacity‐building, sharing power, including diverse voices, valuing people's time and sharing the benefits.^6^, ^7^, ^20^, ^27^

Meaningful codevelopment requires time, funding and infrastructure.^6^, ^21^ We have tried to do justice to this process by reporting our methods in detail. This was particularly true in the context of COVID‐19, where we experienced delays in forming the committee and getting comfortable working together in virtual space. Codevelopment relies on relationship‐building, developing trust and being with communities and partners in shared space.^7^, ^20^, ^45^ It was more difficult to engage in the emerging context of fieldwork over videoconferencing, where committee members did not have an opportunity to gather together, share food and get to know one another in person. Despite this, relationships were built by a shared commitment to improving care, overcoming tensions and an openness to vulnerability. Often, the interactions that supported these relationships happened in the margins—over emails, through encouragement or socialization before and after meetings, and in sharing humour. Participating openly in reflective exercises and sharing personal experiences helped create a trusting environment.^27^ Researcher reflexivity is vital to codevelopment,^20^, ^45^ and K. W. played a key role in identifying power imbalances and promoting researcher self‐reflection.

One component of codevelopment is maintaining ongoing relationships.^21^, ^27^ This article reports findings from the first phase of the committee's involvement in this study. Since the meetings have finished, the committee has continued to meet to produce an infographic and joint conference presentation,^46^ cowrite a report of findings, participate in four workshops with researchers and clinical stakeholders, advise policymakers preparing for an upcoming survey and collaborate on this article.^47^ While this study aims to advance methods in the use of PROMs and PREMs with people who are socially disadvantaged and experience chronic illness, the preliminary nature of this study is unlikely to result in immediate change before further research and policy advocacy. It was important for researchers in this study to be clear about the expected outcomes so as not to raise hopes or make false promises.^14^, ^20^ Despite this, committee members with lived experience identified the value of participating in the codevelopment process as well as the possibility that it could make a positive difference in the lives of people down the line. This echoes research on codevelopment that has found that peer engagement is often driven by a desire to help others.^7^, ^26^

This study's main strength is the people who came together to be involved in it. During an extraordinarily difficult time, committee members with and without experience of codevelopment, research or technology joined, and from the first meeting, had thoughtful questions, insightful feedback and a willingness to challenge assumptions. Other strengths include a codevelopment process that engages people with lived experience through each step of the study,^6^, ^20^, ^21^ the contribution that this study makes as part of an ongoing relationship between researchers and community members in this locale^21^, ^26^ and attention to ethical principles articulated by a similar community of people with lived experience of homelessness and substance use.^27^

There are many barriers to video meetings for people who are socially disadvantaged including needing a secure, private location; an electronic device with a functioning video camera and microphone; time to participate; and internet access. Due to COVID‐19 restrictions, we bypassed some formal community channels and extended direct invitations, a process that is not always recommended.^7^, ^27^ Moll et al.^20^ criticizes invited spaces, raising concerns about tokenism and perpetuating marginalization that may have been reproduced by our small group number. Committee members were in more stable life circumstances than those who may have joined the committee before COVID‐19, and no committee member became homeless during this phase of the study. A different committee composition, or a different format involving a larger group and more targeted discussion topics as originally planned, may have led to different findings.

Both researchers and policymakers stand to benefit from creating meaningful roles for engagement with people with lived experience when designing or selecting PROMs and PREMs. When transferring general‐population PROMs and PREMs to socially disadvantaged groups, it is important to thoughtfully address any underlying assumptions and seek feedback through codevelopment or methods such as cognitive interviewing in addition to traditional validation measures.^21^, ^22^ When selecting and designing PROMs and PREMs for people who are socially disadvantaged, it will also be important to measure experience in the health care system in ways that are sensitive to exposure to stigma and include measures of choice.

## 5 CONCLUSION

This article reports on the findings of the People with Lived Experience Advisory Committee in the first phase of the study ‘Towards Equity‐Informed Care’. Committee discussions aimed to identify and explain what is most important to measure about health care in the context of lived experience of homelessness and chronic illness, and how can it best be measured using PROMs and PREMs. Themes were trust and relationship building; health and quality of life; and equity. Specific recommendations produced by the committee have been presented in Table 1 with the aim of informing PROM and PREM adaptation and implementation for use with people with experience of homelessness and chronic illness.

## AUTHOR CONTRIBUTIONS

Erin Donald, Kara Whitlock and Dr. Kelli Stajduhar contributed to study conception and design in partnership with an existing community advisory team of inner‐city workers and peers who are not involved in the authorship of this article. All authors participated in data collection and analysis. Erin Donald wrote the initial draft of the article and led subsequent revisions. Kara Whitlock worked with Erin Donald to develop concepts and contribute to the discussion and methodology sections. Dr. Kelli Stajduhar provided written feedback on the article. Erin Donald, Kara Whitlock, Tracy Dansereau, Daniel J Sands and David Small met to verbally read through the article, provide feedback and make changes. In total, these meetings accumulated to 8 h of conceptual and technical contribution to the manuscript. The final version including order of authorship was read and approved by all authors before submission.

## CONFLICTS OF INTEREST

The authors have no conflicts of interest.

## Supporting information

Supporting information.

Supporting information.

## Data Availability

Due to committee members' roles as codevelopers (not as participants), anonymized data are not available.
