# Supplementary material for: A codevelopment process to advance methods for the use of patient‐reported outcome measures and patient‐reported experience measures with people who are homeless and experience chronic illness
Source: Health Expect. 2022 Apr 11;25(5):2264–74. doi: 10.1111/hex.13489 (PMC9615092; doi:10.1111/hex.13489)
Supplement: Supplementary file 1 — Supporting information. [file HEX-25--s002.docx]

Appendix A. Lived experience advisory committee document on group process: Guidelines for ethical engagement

May 20, 2020

How we work together: Guidelines for ethical engagement

(Document can be updated as our learning grows)

- Respect is key
- No war stories, keep things specific, don’t go off on long rants
- Have patience
- ‘don’t yuck my yum’.
- Acknowledge ‘ouch’ with ‘oops’. Just go ahead and say ‘ouch’ if someone has said something that’s hurtful.
- There are no dumb questions
- It’s always okay for anyone to say we need to have a break or step away from meeting to have a smoke or go to the bathroom
- Please tell us if anyone is using language that doesn’t sit right
- Use person-first language
