# Supplementary material for: A codevelopment process to advance methods for the use of patient‐reported outcome measures and patient‐reported experience measures with people who are homeless and experience chronic illness
Source: Health Expect. 2022 Apr 11;25(5):2264–74. doi: 10.1111/hex.13489 (PMC9615092; doi:10.1111/hex.13489)
Supplement: Supplementary file 2 — Supporting information. [file HEX-25--s001.docx]

Appendix B. Lived experience advisory committee conceptual discussion prompts

| Concept | Question prompts for research team | Question prompts for Advisory |
| --- | --- | --- |
| Orientation | How do we orient the Advisory Committee (AC) to the project in a way that centers lived expertise when PROM/PREM jargon probably isn’t how people think about their experiences with health care?  How do we describe technical terms in a clear way?  Can we explain the project visually?  Where the advisory work fits in to it?  How do we disrupt the power differentials in our group?  How do we build a relational space online?  How much do we want to share about our personal experiences?  What do we identify as our facilitation goals?  How will we keep discussion focused on the meeting topic yet remain open to the emerging flow?  How do we best identify barriers to engaging with this project in our virtual context? | How should we structure our meetings?  How often should we meet?  How should we share information? Do you want agendas before the meeting or would you rather just show up and talk?  What is important in group process?  How do you want to deal with stipends and payment?  Why is confidentiality important to create an environment of trust? |
| Introduction | How do we bridge the gaps in our knowledge to address the research question?  How do we take the role of learners as well? How might we demonstrate this?  How do we know we have reached a mutual understanding?  What does transparency look like in this work?  How do I identify the tension of being both a person with lived experience as well as a researcher.  How do I feel about this duality? | How do we work together in a way that holds space for difference?  What do we need to know about each other to do this work?  Who are you and what would you like us to know about you?  What would you like to know about us?  How do we make room for questions and clarification? |
| Care |  | What is good health care?  what does good health care look like?  How is good care demonstrated?  Has your perception of good care changed over time?  What are your goals of care?  What do researchers need to know about this population and care? |
| Relationship | Is health care a relationship?  Is this relationship different depending on your access to resources and choice? | What is trust in health care?  How do you develop relationship with your health providers?  Do you have examples that demonstrated relationship?  How would you measure this relationship quality? |
| QoL |  | What does quality of life mean to you?  What does QoL look like?  How would you measure it? |
| Challenges and strategies in health care |  | What are some challenges for the community in getting care?  What are the strategies you have used to get health needs met?  What happens when you tell the truth?  What do researchers need to know about these challenges? |
| Measurement | How might we use the terms and concepts defined in advisory work to describe measurement tools?  How do we share enough information for meaningful participation without prioritizing researcher expertise (even unintentionally)? | Let’s brainstorm what would work to capture experiences.  What impacts the decision to seek care for folks?  Picking measures: how do we start looking at specific tools? |
| When examining individual tools |  | What do you like about this tool? What don’t you like?  Do you think this tool would work in practice with people who have chronic illnesses and have experienced homelessness? Why or why not?  What would you change about this tool? What would you want to see in a tool designed to capture your experiences and outcomes? |
| Planning our workshop | How do we attend to power differences when we bring the two groups together (the advisory and the research team)?  How do we organize our discussions into workshops? | How do we want to meet with the research team and how might we disrupt the power dynamics in the room?  What is important to share in the workshop about our discussions?  Who wants to do what during the workshop? |
